# Investigation of opioid use and long-term oncologic outcomes for non-small cell lung cancer patients treated with surgery

**DOI:** 10.1371/journal.pone.0181672

**Published:** 2017-07-21

**Authors:** Tak Kyu Oh, Jae Hyun Jeon, Jong Mog Lee, Moon soo Kim, Jee Hee Kim, Hyeyeon Cho, Seong-eun Kim, Woosik Eom

**Affiliations:** 1 Department of Anesthesiology and Pain Medicine, Seoul National University Bundang Hospital, Seongnam, Gyeonggi-do, Republic of Korea; 2 Department of Thoracic Surgery, National Cancer Center, 323, Ilsan-ro, Ilsandong-gu, Goyang-si, Gyeonggi-do, Republic of Korea; 3 Department of Anesthesiology and Pain Medicine, National Cancer Center, 323, Ilsan-ro, Ilsandong-gu, Goyang-si, Gyeonggi-do, Republic of Korea; 4 Department of Neurology, Seoul National University Bundang Hospital, Seongnam, Gyeonggi-do, Republic of Korea; Temple University, UNITED STATES

## Abstract

Opioids are commonly used for postoperative pain control in cancer patients. In addition to pain control, an association between opioid use and long-term oncologic outcomes, such as recurrence or overall survival, has been postulated. The aim of this study was to determine whether postoperative opioid use in patients with non-small cell lung cancer is associated with long-term oncologic outcomes, including recurrence and death. Data obtained from 1009 medical records of patients who underwent curative resection at the National Cancer Center, Korea between January 2006 and December 2010 were retrospectively analyzed. Seven-day opioid use was divided into four quartiles to analyze probability of recurrence and death. Multivariate regression analyses of recurrence and death was conducted, including the calculation of odds ratios. A total of 871 patients were analyzed. When opioid dosage was examined by quartiles, the probability of death and recurrence increased gradually with increasing opioid use. However, in the multivariate regression analysis, the amount of opioid usage did not affect the risk of recurrence or death of lung cancer (*P* = 0.520 for recurrence; *P* = 0.659 for death). Opioid use was correlated with outcome when stratified by lung cancer stage (*P* = 0.004 for recurrence; *P* = 0.049 for death); however, the odds ratios only slightly increased (1.001 for stage IA–IIIA) for both outcomes. In non-small cell lung cancer patients, the amount of opioid usage does not affect the risk of recurrence and death of lung cancer. There was an association with stage (IA–IIIA), but the effect was negligible. A well-designed prospective study is needed.

## Introduction

Lung cancer is one of the most common cancers worldwide. Non-small cell lung cancer (NSCLC) accounts for approximately 80% of all diagnosed lung cancers [1]. Surgical management is a potentially curative option that can ensure long-term survival of patients with early stage NSCLC. Even in selected patients with advanced stage NSCLC, such as N2 disease, multimodal treatment including surgical resection is performed with curative intent [2]. However, even after curative resection of NSCLC, long-term survival is observed in less than 50% of patients, and recurrence occurs in 33.1% of patients within the median 2-year follow-up [3].

Opioids are the most frequently used analgesics during the intraoperative and postoperative periods. Opioids reduce the activity of natural killer (NK) cells and may contribute to the recurrence and metastasis of cancer cells through immunosuppression [4]. In addition, opioids exhibit pro-tumoral effects that enhance cancer cell growth via vascular endothelial growth factor [5, 6]. Based on these past reports, retrospective studies have been conducted to analyze the relationship between opioid use in patients with NSCLC and cancer recurrence rates. Studies have reported an association between cancer recurrence and the variation in the dosage of intraoperative and postoperative opioids [7, 8]. However, the precise effects of opioids on cancer recurrence are still controversial in clinical practice [9, 10]. Therefore, it is necessary to study whether postoperative opioid use is associated with long-term oncologic outcomes such as recurrence or death.

In this study, we aimed to investigate the difference in postoperative recurrence and survival in NSCLC patients according to opioid usage. We hypothesized that opioid use would increase the risk of recurrence or death in NSCLC patients according to stage.

## Methods

### Patient data

This retrospective cohort analysis was approved by the Institutional Review Board of the National Cancer Center in Korea (NCC2015-0297). Electronic medical records of patients diagnosed with NSCLC who underwent elective surgical resection between January 1, 2006 and December 31, 2010 were used for the analysis. Patients aged 18–85 years with a final pathological diagnosis of stage IA, IB, IIA, IIB or IIIA NSCLC who underwent a lobectomy, bilobectomy, or sleeve lobectomy were included in this study. A single-lobe resection with additional sublobar resection was considered a lobectomy. The exclusion criteria were as follows: 1) death from postoperative complications within 1 month of surgery, 2) the need for revision surgery within 1 week of surgery, 3) incomplete electronic medical records with respect to opioid use, 4) occurrence of a different primary cancer within 5 years of surgery, 5) loss to follow-up within 5 years after surgery, 6) intraoperative conversion to pneumonectomy or sublobar resection, and 7) preoperative opioid use.

The following patient data were collected: gender, age, height, weight, preoperative forced expiratory volume 1, Charlson Comorbidity Index score, histological tumor type, recurrence type, smoking history, neoadjuvant and adjuvant chemotherapy, American Society of Anesthesiologists score, operation type, epidural analgesia, and cancer stage, including tumor, lymph node, and metastasis stage based on the American Joint Committee on Cancer 7th cancer staging system. Based on a previous study, recurrence in the surgical resection margin, ipsilateral hilum, and/or mediastinum was classified as locoregional recurrence, and all other recurrences involving the contralateral hilum and supraclavicular fossa were considered distant metastases [11]. Anatomic pulmonary resection and mediastinal lymph node dissection were performed as either minimally invasive surgery (video-assisted thoracic surgery [VATS] or Robot-assisted) or standard posterolateral thoracotomy. Minimally invasive surgery was performed under direct monitor-vision without rib spreading. Standard posterolateral thoracotomy was performed by the same surgical team after dividing the latissimus dorsi muscle and preserving the serratus anterior muscle. In addition, anesthesiologists administered sevoflurane as the surgical inhalation anesthetic using a standardized anesthetic technique. During surgery, continuous infusion of opioids, including remifentanil, was not performed.

As patients who underwent a lobectomy were discharged from the hospital after a minimum of 6 days following the surgery at the National Cancer Center in Korea, data of intraoperative and 6-day postoperative opioid use were combined. In general, patients used patient-controlled analgesia (PCA) until postoperative day (POD) 3. When using the epidural PCA, 0.1875% ropivacaine and 20 mg of epidural morphine were used. For intravenous PCA, 100 mL or 200 mL mixed regimens that included 0.5 mg of morphine and 15 μg of fentanyl per mL were used. During and after use of epidural PCA and intravenous PCA, additional intravenous and oral opioids were used depending on the patient’s complaints of pain. These amounts were converted to equianalgesic doses of oral morphine by using a standardized conversion ratio and then combined for the total opioid dose (S1 Table) [12, 13].

The primary outcome of this study was recurrence or death after surgery in each stage of NSCLC. All data were fully anonymized by medical records technicians at the National Cancer Center, Korea, and the Institutional Review Board waived the requirement for informed consent due to the retrospective study design.

### Statistical analysis

To review any relationship between each covariate and opioid usage, we performed a univariate analysis. In that analysis, we applied one-way ANOVA for continuous covariates and Pearson’s chi-square test for categorical covariates on opioid use categorized by quartiles. From the previous analysis, sets of covariates satisfying *P* < 0.1 were selected and included in the multivariate model for adjustment. We used multivariate logistic regression analysis with consideration for a clinically plausible interaction to estimate the association of opioid usage with recurrence of lung cancer and death due to lung cancer. In the multivariate logistic regression model, we adjusted for the selected covariates from the univariate analysis. Throughout the analysis, *P* < 0.05 was considered statistically significant.

## Results

In total, 1009 patients with NSCLC met the inclusion criteria for this study. A further 138 patients were excluded from the final analysis of the study, including 25 who died of postoperative complications within 1 month of surgery, 10 who underwent incomplete resection, 28 who were diagnosed with additional primary cancer in other locations within 5 years after surgery, 35 who were lost to follow-up within 5 years of surgery, 2 who underwent an intraoperative conversion to pneumonectomy, and 38 with incomplete medical records. As a result, 871 patients were included in the final analyses.

Table 1 gives information about all of the covariates for opioid usage categorized by quartiles [Q1 (first quartile) = 819.0; Q2 (median) = 1359.6; Q3 (third quartile) = 2148.0], including their differences. Among the sets of covariates selected from the univariate analysis in Table 1 satisfying p-values less than 0.1, only clinically important covariates were considered in the multivariate logistic regression model. The following variables were included in the multivariate logistic regression model: gender, age, height, weight, Charlson Comorbidity Index score, histological type, receipt of adjuvant chemotherapy, operation type I (lobectomy, bilobectomy, and sleeve lobectomy), operation type II (VATS or Robot-assisted and Open thoracotomy), receipt of epidural analgesia, tumor stage, and nodal involvement.

**Table 1:** Demographics and baseline characteristics for opioid groups by quartiles.

Variable		Opioid < Q1	Q1 ≤ Opioid < Q2	Q2 ≤ Opioid < Q3	Opioid ≥ Q3	*P*-value
		N = 213	N = 222	N = 218	N = 218	
Gender (%)						< .0001
	Male	122 (57.28)	167 (75.23)	167 (76.61)	172 (78.90)	
	Female	91 (42.72)	55 (24.77)	51 (23.39)	46 (21.10)	
Age (years)						0.1596
	Mean (SD)	62.75 (9.76)	63.51 (8.69)	62.12 (9.19)	61.58 (9.86)	
Height (cm)						< 0.0001
	Mean (SD)	161.2 (8.17)	164.1 (7.10)	163.8 (7.93)	164.2 (7.30)	
Weight (kg)						0.0007
	Mean (SD)	60.36 (9.91)	63.72 (9.57)	63.84 (10.68)	62.99 (9.67)	
Preoperative FEV1 (L)						0.7223
	Mean (SD)	2.35 (0.61)	2.36 (0.63)	2.36 (0.62)	2.41 (0.63)	
Charlson Comorbidity Index score						0.0024
	Mean (SD)	2.25 (0.49)	2.45 (0.67)	2.44 (0.68)	2.40 (0.59)	
Type of Recurrence (%)						< 0.0001
	No recurrence	159 (34.05)	128 (27.41)	93 (19.91)	87 (18.63)	
	Locoregional	13 (12.26)	22 (20.75)	31 (29.25)	40 (37.74)	
	Distant metastasis	41 (13.76)	72 (24.16)	94 (31.54)	91 (30.54)	
Histological type (%)						0.0044
	SqCC	67 (31.46)	104 (46.85)	99 (45.41)	95 (43.58)	
	AdenoCA	127 (59.62)	105 (47.30)	96 (44.04)	98 (44.95)	
	Other NSCLCs	19 (8.92)	13 (5.86)	23 (10.55)	25 (11.47)	
History of Smoking (%)						0.0005
	Yes	122 (57.28)	158 (71.17)	154 (70.64)	163 (74.77)	
	No	91 (42.72)	64 (28.83)	64 (29.36)	55 (25.23)	
Neoadjuvant CTx (%)						0.2020
	Yes	10 (4.69)	5 (2.25)	4 (1.83)	10 (4.59)	
	No	203 (95.31)	217 (97.75)	214 (98.17)	208 (95.41)	
Adjuvant CTx (%)						< .0001
	Yes	53 (24.88)	88 (39.64)	88 (40.37)	106 (48.62)	
	No	160 (75.12)	134 (60.36)	130 (59.63)	112 (51.38)	
ASA class (%)						0.0486
	I	70 (32.86)	49 (22.07)	49 (22.48)	55 (25.23)	
	II	138 (64.79)	164 (73.87)	155 (71.10)	156 (71.56)	
	III	5 (2.35)	9 (4.05)	14 (6.42)	7 (3.21)	
Operation Type I (%)						0.1221
	Lobectomy	192 (25.98)	188 (25.44)	183 (24.76)	176 (23.82)	
	Bilobectomy	11 (15.49)	18 (25.35)	16 (22.54)	26 (36.62)	
	Sleeve lobectomy	10 (16.39)	16 (26.23)	19 (31.15)	16 (26.23)	
Operation Type II (%)						< .0001
	VATS or robot	110 (51.64)	95 (42.79)	69 (31.65)	62 (28.44)	
	Open	103 (48.36)	127 (57.21)	149 (68.35)	156 (71.56)	
Combined Epidural Anesthesia (%)						< .0001
	Yes	134 (62.91)	142 (63.96)	148 (67.89)	207 (94.95)	
	No	79 (37.09)	80 (36.04)	70 (32.11)	11 (5.05)	
Stage (%)						< .0001
	IA	73 (39.25)	53 (28.49)	34 (18.28)	26 (13.98)	
	IB	54 (24.00)	55 (24.44)	60 (26.67)	56 (24.89)	
	IIA	38 (25.68)	39 (26.35)	35 (23.65)	36 (24.32)	
	IIB	15 (15.79)	26 (27.37)	26 (27.37)	28 (29.47)	
	IIIA	33 (15.21)	49 (22.58)	63 (29.03)	72 (33.18)	
Tumor (%)						0.0026
	T1a	55 (36.18)	42 (27.63)	25 (16.45)	30 (19.74)	
	T1b	31 (30.39)	27 (26.47)	27 (26.47)	17 (16.67)	
	T2a	82 (22.97)	87 (24.37)	94 (26.33)	94 (26.33)	
	T2b	22 (22.22)	25 (25.25)	20 (20.20)	32 (32.32)	
	T3	22 (14.57)	39 (25.83)	48 (31.79)	42 (27.81)	
	T4	1 (0.00)	2 (0.01)	4 (0.02)	3 (0.01)	
Node (%)						0.0003
	N0 or N1	154 (72.30)	142 (63.96)	125 (57.34)	117 (53.67)	
	N2	35 (16.43)	51 (22.97)	54 (24.77)	47 (21.56)	
	N3	24 (11.27)	29 (13.06)	39 (17.89)	54 (24.77)	

*P*-value by One-Way ANOVA or Pearson's chi-square test as appropriate.

Other NSCLCs: Large cell type, Sarcomatoid type

Q1 (the first quartile) = 819.0; Q2 (median) = 1359.6; Q3 (the third quartile) = 2148.0

SD, Standard Deviation; FEV1, forced expiratory volume 1; SqCC, Squamous Cell Carcinoma; NSCLC, Non-Small Cell Lung Cancer; CTx, Chemotherapy; ASA, American Society of Anesthesiologists; VATS, Video-Assisted Thoracic surgery; SqCC: squamous cell carcinoma; AdenoCA, adenocarcinoma

**Fig 1:**
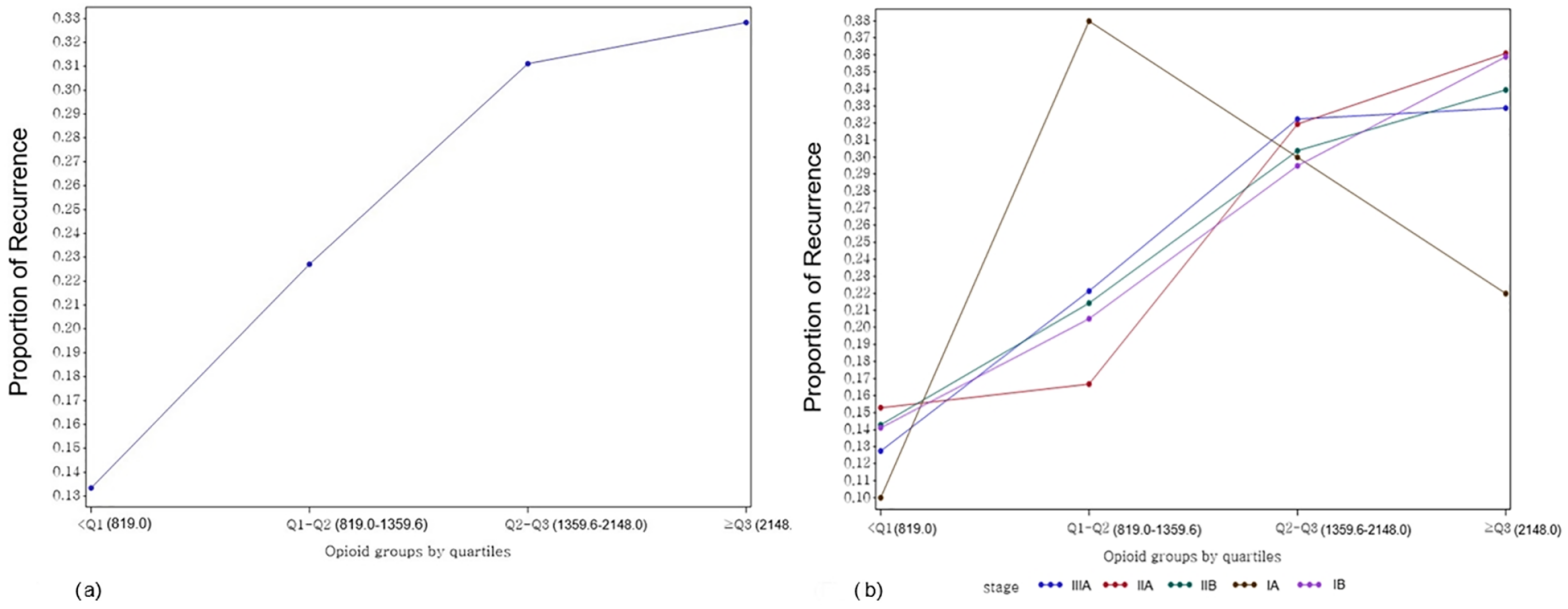
**Proportions of overall (a) and stage-specific (b) recurrence in opioid usage groups by quartiles.** Q1 (first quartile) = 819.0; Q2 (median) = 1359.6; Q3 (third quartile) = 2148.0.

**Fig 2:**
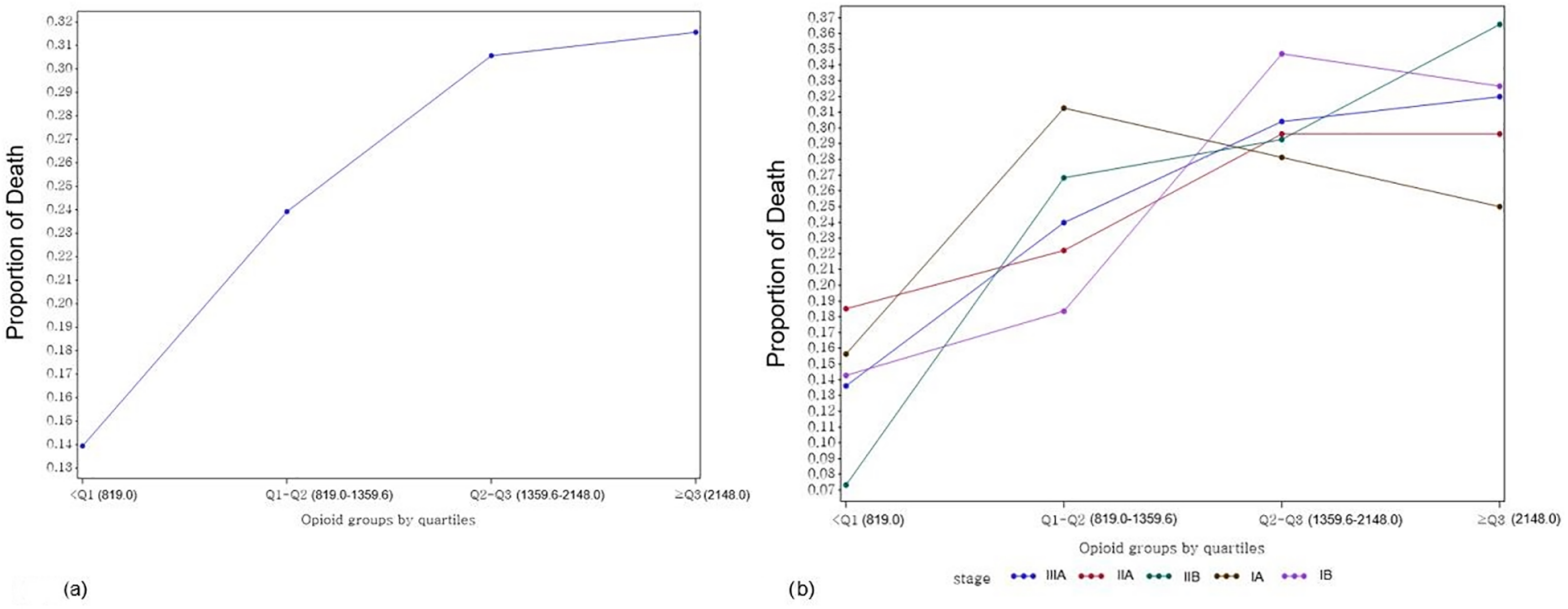
**Proportions of overall (a) and stage-specific (b) death on opioid usage groups by quartiles.** Q1 (first quartile) = 819.0; Q2 (median) = 1359.6; Q3 (third quartile) = 2148.0.

Without adjusting for the selected covariates, we examined the proportions of recurrence and death over all stages of lung cancer (Figs 1A, 1B, 2A and 2B). Regardless of stage, the proportions of recurrence and death increased with higher opioid usage. However, for stage Ia, the proportions of both recurrence and death showed extreme variations (Figs 1B and 2B).

Table 2 shows that the amount of opioid usage did not affect the risk of either recurrence or death from lung cancer (*P* = 0.5206 for recurrence; *P* = 0.6597 for death). However, when analyzed according to lung cancer stage, the effect of opioids was correlated to outcome (*P* = 0.004 for recurrence; *P* = 0.049 for death). Table 3 presents the odds ratios (ORs) of recurrence and death for 1 unit of opioid usage increase across all stages of lung cancer. While the ORs for the recurrence of lung cancer at stages IA, IB, and IIA were significantly different with a 1-unit opioid usage increase, the values were barely over 1.001 at 1.002. We might conclude there is no risk of recurrence or death from lung cancer caused by opioid usage across all stages. Similarly, the ORs of death due to lung cancer might not be affected by opioid usage for all stages.

**Table 2:** Parameters estimated in the multivariate logistic regression model.

Recurrence				Death			
Effect	DF	Wald Chi-Square	P-value	Effect	DF	Wald Chi-Square	P-value
Opioid Dose	1	0.4128	0.520	Opioid Dose	1	0.1939	0.659
Gender	1	0.7596	0.383	Gender	1	1.4835	0.223
**Age (years)**	1	14.1015	**< 0.001**	**Age (years)**	1	11.2553	**< 0.001**
Weight (kg)	1	3.2820	0.070	**Weight (kg)**	1	6.5298	0.010
Height (cm)	1	0.0778	0.780	Height (cm)	1	0.8672	0.351
**Charlson Comorbidity Index score**	1	12.5445	**< 0.001**	**Charlson Comorbidity Index score**	1	23.3209	**< 0.0001**
Histological type	2	2.1475	0.341	Histological type	2	2.6095	0.271
**Adjuvant CTx**	1	4.8515	**0.027**	**Adjuvant CTx**	1	11.3244	**< 0.001**
Operation type I	2	0.5686	0.752	Operation type I	2	1.1539	0.561
Operation type II	1	1.2505	0.263	Operation type II	1	0.0898	0.764
Epidural	1	1.9090	0.167	**Epidural**	1	4.2208	**0.039**
Stage	4	5.8312	0.212	**Stage**	4	18.6972	**< 0.001**
**Tumor**	5	13.3037	**0.020**	Tumor	5	7.5819	0.180
**Node**	2	8.2435	**0.016**	Node	2	0.7993	0.670
Dose*Stage	4	15.1334	**0.004**	Dose*Stage	4	9.5378	**0.049**

Operation Type I: Lobectomy, Bilobectomy, and Sleeve Lobectomy, Operation Type II: Video-Assisted Thoracic Surgery, Robot-Assisted or Open Thoracotomy, CTx, Chemotherapy

**Table 3:** Odds ratios of recurrence and death for 1 unit opioid usage increase over all cancer stage.

Recurrence					Death				
Odds Ratio	Estimate	95% Confidence Limits		P-value	Odds Ratio	Estimate	95% Confidence Limits		P-value
**Stage IA**	1.001	1.001	1.001	0.0041	**Stage IA**	1.001	1.000	1.001	0.0105
**Stage IB**	1.001	1.000	1.001	0.0243	**Stage IB**	1.001	1.000	1.001	0.0317
**Stage IIA**	1.001	1.001	1.002	0.0006	**Stage IIA**	1.000	1.000	1.001	0.1774
**Stage IIB**	1.001	1.000	1.001	0.1210	**Stage IIB**	1.001	1.000	1.001	0.0363
**Stage IIIA**	1.000	1.000	1.000	0.5206	**Stage IIIA**	1.000	1.000	1.000	0.6597

**Fig 3:**
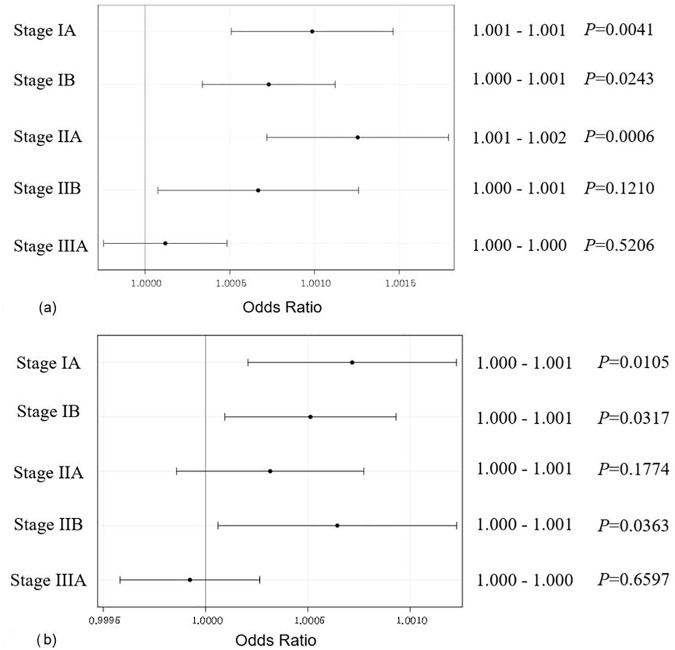
**Odds ratios of recurrence (a) and death (b) for 1 unit opioid usage increment over all the stages**.

**Fig 4:**
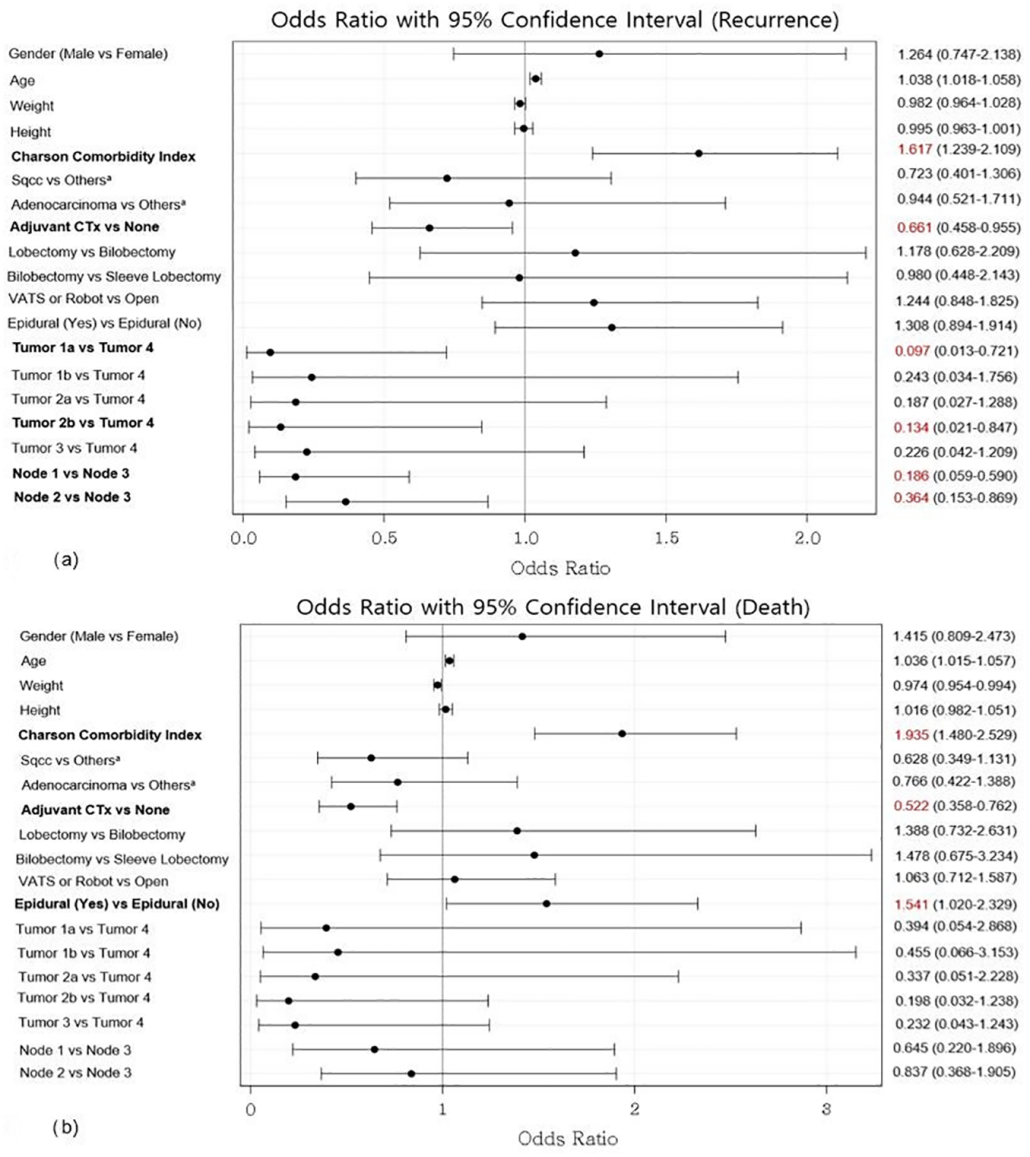
**Odds ratios of recurrence (a) and death (b) for all covariates.** Others*: Large cell type, sarcomatoid type; CTx, Chemotherapy; VATS, Video-Assisted Thoracic Surgery.

Fig 3A and 3B show the estimated ORs of recurrence and death across all stages after adjusting the covariates in the model. Variables related to recurrence were the Charlson Comorbidity Index score, adjuvant chemotherapy, tumor stage, and node stage (Fig 4A). The variables related to the risk of death were the Charlson Comorbidity Index score, adjuvant chemotherapy, and epidural analgesia (Fig 4B).

## Discussion

Our study showed that opioid use was not an independent risk factor for either recurrence or death in patients with NSCLC. Moreover, despite opioid use showing correlation to outcome when stratified by cancer stage, the ORs were close to 1 (1.000–1.001) for all covariates. Our study showed negative results regarding the association of opioid use after lung cancer surgery with recurrence or death. This is consistent with a previous study that opioid use during surgery in lung cancer is not associated with recurrence-free survival [7]. Furthermore, our study was characterized by the use of multivariate regression analysis to calculate all opioid use for a total of 7 days up to POD 0–6, which is a relatively long period of opioid use compared to previous studies [7, 8].

The lung contains the largest percentage of NK cells among the peripheral organs [14], and the postoperative period is known as the ‘decisive period,’ when NK cell-mediated immunity suppresses emerging lung cancer metastasis [15, 16]. Based on these assumptions, a previous study by Maher et al. [8] reported that a group of 99 recurrent patients who underwent VATS lung cancer surgery had greater postoperative opioid use than the non-recurrent group. Interestingly, this study also found an association between total opioid use and lung cancer recurrence with a hazard ratio of 1.003 (95% CI 1.000–1.006, *P* = 0.04), similar to that seen in our study [8]. The differences between our study and that by Maher et al. [8] are that we had a higher percentage of open thoracotomy than VATS, calculated the opioid usage over a longer postoperative period of time (POD 0–6), and included more advanced stages (up to stage IIIA). Despite these differences, the final ORs were similar between the two studies.

An important issue in our study was the relatively high opioid dose administered (median: 1359.6 mg of oral morphine equivalent). The administration of opioids in the study by Maher et al. [8] up to 96 hours post-surgery was 124 mg in the non-recurrence group and 232 mg in the recurrence group, which was much lower than that in our study. There are three reasons for this. First, our study included only patients who underwent surgery between 2006 and 2010 and were followed-up for at least 5 years. During this period, our hospital had a higher rate of open thoracotomy than VATS. In fact, 61.4% (535/871) of patients underwent open thoracotomy. Therefore, direct comparisons with the study of Maher et al. [8], who included only VATS patients, is challenging. A second consideration is that our study covered the use of opioids during the intraoperative period and the 6-day postoperative period, for a total of 7 days of hospitalization. This is a much longer time period (168 hours) than those in other studies [7, 8], such as the study by Maher et al., which was limited to 96 hours. Furthermore, anesthesiologists in our hospital actively used opioids for “balanced anesthesia” during surgery to effectively anesthetize patients, since thoracotomy is accompanied by severe pain. Finally, we calculated the opioid injected through the epidural route by adding it to the intravenous opioids. This was based on the result of a previous study in which epidural opioids were eventually removed by systemic circulation through Cerebrospinal fluid [17]. Our study has attempted to include all operations performed with open thoracotomy (bilobectomy, sleeve lobectomy, lobectomy) for a relatively longer period than previous studies. Therefore, relatively more opioids were administered than in other studies.

Postoperative complications are another important issue, as their absence can result in improved long-term survival after cancer surgery [18]. Although a negative effect of postoperative complications on cancer recurrence was observed in patients with esophageal cancer [19], the direct relationship between recurrence and postoperative complications is still controversial. In our study, patients who experienced surgical complications used more opioids compared to those who did not, which could be a confounding factor. To avoid any effects from this confounding factor, we excluded patients who died of postoperative complications within 1 month. However, further prospective studies are needed to clarify the relationship between postoperative complications and cancer recurrence.

Finally, this study examined the effect of opioids on lung cancer stage. In previous studies [7, 8], opioid use was associated with long-term oncologic outcome in early stage NSCLC, with our study showing fewer correlations in stage IIB and IIIA. However, another study reported that opioid use was an independent predictor of survival in advanced NSCLC patients [20]. Although this study was not in the postoperative setting [20], it remains unclear whether the use of opioids is clinically relevant to patients with NSCLC. Therefore, further studies are needed.

Our study has several limitations. First, as a retrospective study, there were patients with incomplete medical records who had to be excluded from the final analysis. Second, opioids were administered via three different methods (intravenous, epidural, and oral), requiring a conversion of oral morphine to equianalgesic doses. Moreover, the accuracy of this conversion ratio is unclear, especially with the use of opioids with longer biological half-lives or higher dosages [21, 22]. Furthermore, although epidural opioids are removed from the patient’s cerebrospinal fluid via the systemic circulation [17], there is no accurate study comparing the pro-tumoral effects of epidural and systemic opioids. Third, as mentioned above, postoperative complications were not considered as factors affecting recurrence after surgery. Fourth, a follow-up time of 5 years could be insufficient to evaluate the risk of recurrence after surgery. Finally, there was a lack of homogeneity in the target population, and there could have been some confounding factors, such as aggressive tumors and extensive resection (e.g., chest wall and sleeve), which could have resulted in unavoidable selection bias. However, this allowed us to analyze not only patients with early-stage NSCLC, but also those with advanced stage disease, as well as those who underwent open thoracotomies. Despite these limitations, our study is one of the first studies, to the best of our knowledge, to show an effect of postoperative opioid use on recurrence and survival based on NSCLC stage (IA–IIIA).

In conclusion, intraoperative and postoperative use of opioid does not affect the risk of recurrence or death due to lung cancer. There was also an association with stage (IA–IIIA), but the effect was negligible. A well-designed prospective study is needed to examine the effect of decreasing opioid use on long-term oncologic outcome in patients with NSCLC.

## Supporting information

S1 Table Equianalgesic opioid conversion table. All equianalgesic doses are given relative to the equivalent dose of 10 mg of oral morphine.
(DOCX)

## References

[1] Jemal A, Bray F, Center MM, Ferlay J, Ward E, Forman D. Global cancer statistics. CA Cancer J Clin. 2011;61: 69–90. doi: 10.3322/caac.20107 21296855 10.3322/caac.20107

[2] Pearson FG. Non-small cell lung cancer: role of surgery for stages I-III. Chest. 1999;116: 500S–503S. 10619519 10.1378/chest.116.suppl_3.500s

[3] Taylor MD, Nagji AS, Bhamidipati CM, Theodosakis N, Kozower BD, Lau CL, et al Tumor recurrence after complete resection for non-small cell lung cancer. Ann Thorac Surg. 2012;93: 1813–1820; discussion 1820–1821. doi: 10.1016/j.athoracsur.2012.03.031 22542070 10.1016/j.athoracsur.2012.03.031

[4] Cata JP, Gottumukkala V, Sessler DI. How regional analgesia might reduce postoperative cancer recurrence. Eur J Pain Suppl. 2011;5: 345–355.

[5] Lennon FE, Moss J, Singleton PA. The μ-opioid receptor in cancer progression: is there a direct effect? Anesthesiology. 2012;116: 940–945. doi: 10.1097/ALN.0b013e31824b9512 22357347 10.1097/ALN.0b013e31824b9512

[6] Mathew B, Lennon FE, Siegler J, Mirzapoiazova T, Mambetsariev N, Sammani S, et al The novel role of the mu opioid receptor in lung cancer progression: a laboratory investigation. Anesth Analg. 2011;112: 558–567. doi: 10.1213/ANE.0b013e31820568af 21156980 10.1213/ANE.0b013e31820568af PMC4327979

[7] Cata JP, Keerty V, Keerty D, Feng L, Norman PH, Gottumukkala V, et al A retrospective analysis of the effect of intraoperative opioid dose on cancer recurrence after non-small cell lung cancer resection. Cancer Med. 2014;3: 900–908. doi: 10.1002/cam4.236 24692226 10.1002/cam4.236 PMC4303157

[8] Maher DP, Wong W, White PF, McKenna R Jr., Rosner H, Shamloo B, et al Association of increased postoperative opioid administration with non-small-cell lung cancer recurrence: a retrospective analysis. Br J Anaesth. 2014;113: i88–94. doi: 10.1093/bja/aeu192 25009195 10.1093/bja/aeu192

[9] Bharati SJ, Chowdhury T, Bergese SD, Ghosh S. Anesthetics impact on cancer recurrence: What do we know? J Cancer Res Ther. 2016;12: 464–468. doi: 10.4103/0973-1482.148670 27461594 10.4103/0973-1482.148670

[10] Byrne K, Levins KJ, Buggy DJ. Can anesthetic-analgesic technique during primary cancer surgery affect recurrence or metastasis? Can J Anaesth. 2016;63: 184–192. doi: 10.1007/s12630-015-0523-8 26497721 10.1007/s12630-015-0523-8

[11] Kelsey CR, Marks LB, Hollis D, Hubbs JL, Ready NE, D'Amico TA, et al Local recurrence after surgery for early stage lung cancer: an 11-year experience with 975 patients. Cancer. 2009;115: 5218–5227. doi: 10.1002/cncr.24625 19672942 10.1002/cncr.24625

[12] Benzon H, Rathmell JP, Wu CL, Turk DC, Argoff CE. Raj's practical management of pain 4th ed. Philadelphia: Elsevier Health Sciences; 2008.

[13] Jacox A, Carr D, Payne R, Berde C, Breitbart W, Cain J, et al Management of cancer pain: adults. Cancer Pain Guideline Panel. Agency for Health Care Policy and Research. Am Fam Physician. 1994;49: 1853–1868. 8203323

[14] Hesker PR, Krupnick AS. The role of natural killer cells in pulmonary immunosurveillance. Front Biosci (Schol Ed). 2013;5: 575–587. 23277070 10.2741/s391 PMC4413461

[15] Biki B, Mascha E, Moriarty DC, Fitzpatrick JM, Sessler DI, Buggy DJ. Anesthetic technique for radical prostatectomy surgery affects cancer recurrence: a retrospective analysis. Anesthesiology. 2008;109: 180–187. doi: 10.1097/ALN.0b013e31817f5b73 18648226 10.1097/ALN.0b013e31817f5b73

[16] Exadaktylos AK, Buggy DJ, Moriarty DC, Mascha E, Sessler DI. Can anesthetic technique for primary breast cancer surgery affect recurrence or metastasis? Anesthesiology. 2006;105: 660–664. 17006061 10.1097/00000542-200610000-00008 PMC1615712

[17] Sjöström S, Hartvig P, Persson MP, Tamsen A. Pharmacokinetics of epidural morphine and meperidine in humans. Anesthesiology. 1987;67: 877–888. 2891328 10.1097/00000542-198712000-00002

[18] Law WL, Choi HK, Lee YM, Ho JW. The impact of postoperative complications on long-term outcomes following curative resection for colorectal cancer. Ann Surg Oncol. 2007;14: 2559–2566. doi: 10.1245/s10434-007-9434-4 17522945 10.1245/s10434-007-9434-4

[19] Lerut T, Moons J, Coosemans W, Van Raemdonck D, De Leyn P, Decaluwe H, et al Postoperative complications after transthoracic esophagectomy for cancer of the esophagus and gastroesophageal junction are correlated with early cancer recurrence: role of systematic grading of complications using the modified Clavien classification. Ann Surg. 2009;250: 798–807. doi: 10.1097/SLA.0b013e3181bdd5a8 19809297 10.1097/SLA.0b013e3181bdd5a8

[20] Zylla D, Kuskowski MA, Gupta K, Gupta P. Association of opioid requirement and cancer pain with survival in advanced non-small cell lung cancer. Br J Anaesth. 2014 doi: 10.1093/bja/aeu351 25303989 10.1093/bja/aeu351 PMC6223789

[21] O'Bryant CL, Linnebur SA, Yamashita TE, Kutner JS. Inconsistencies in opioid equianalgesic ratios: clinical and research implications. J Pain Palliat Care Pharmacother. 2008;22: 282–90. doi: 10.1080/15360280802537241 21923312 10.1080/15360280802537241

[22] Patanwala AE, Duby J, Waters D, Erstad BL. Opioid conversions in acute care. Ann Pharmacother. 2007;41: 255–266. doi: 10.1345/aph.1H421 17299011 10.1345/aph.1H421

