# Supplementary material for: Investigation of opioid use and long-term oncologic outcomes for non-small cell lung cancer patients treated with surgery
Source: PLoS One. 2017 Jul 21;12(7):e0181672. doi: 10.1371/journal.pone.0181672 (PMC5521798; doi:10.1371/journal.pone.0181672)
Supplement: S1 Table — All equianalgesic doses are given relative to the equivalent dose of 10 mg of oral morphine. (DOCX) [file pone.0181672.s001.docx]

Supporting Information 1. Equianalgesic opioid conversion table. All equianalgesic doses are given relative to the equivalent dose of 10 mg of oral morphine.

| Opioid | Administration route | Dose equivalent to 10 mg of oral morphine (mg) |
| --- | --- | --- |
| Morphine | Oral | 10 |
| Morphine | I.V. | 3.3 |
| Morphine | Epidural | 0.33 |
| Hydromorphone | Oral | 2 |
| Fentanyl | I.V | 0.03 |
| Oxycodone | Oral | 7 |
| Codeine | Oral | 80 |
| Tramadol | Oral | 40 |
